# Impact of Long Noncoding RNA LINC00673 Genetic Variants on Susceptibility to Diabetic Retinopathy

**DOI:** 10.3389/fgene.2022.889530

**Published:** 2022-04-25

**Authors:** Chih-Chun Chuang, Yi-Sun Yang, Edy Kornelius, Chien-Ning Huang, Min-Yen Hsu, Chia-Yi Lee, Shun-Fa Yang

**Affiliations:** ^1^ Institute of Medicine, Chung Shan Medical University, Taichung, Taiwan; ^2^ Department of Ophthalmology, Changhua Christian Hospital, Changhua, Taiwan; ^3^ School of Medicine, Chung Shan Medical University, Taichung, Taiwan; ^4^ Department of Internal Medicine, Division of Endocrinology and Metabolism, Chung Shan Medical University Hospital, Taichung, Taiwan; ^5^ Department of Ophthalmology, Chung Shan Medical University Hospital, Taichung, Taiwan; ^6^ Department of Ophthalmology, Nobel Eye Institute, Taipei, Taiwan; ^7^ Department of Medical Research, Chung Shan Medical University Hospital, Taichung, Taiwan

**Keywords:** long intergenic noncoding RNA 00673, polymorphism, diabetic retinopathy, diabetes mellitus, disease duration

## Abstract

Long noncoding RNAs (lncRNAs) have been proven to play critical roles in diabetic retinopathy (DR). This study investigated whether the single nucleotide polymorphism (SNP) of long intergenic noncoding RNA 00673 (*LINC00673*) affects the clinical characteristics of diabetic retinopathy (DR). A total of three loci of *LINC00673* SNPs (rs6501551, rs9914618, and rs11655237) were genotyped using TaqMan allelic discrimination in 276 and 454 individuals with and without DR, respectively. Our results revealed that *LINC00673* SNP rs11655237 CT genotype (AOR: 1.592, 95% CI: 1.059–2.395, *p* = 0.026), CT + TT genotype (AOR: 1.255, 95% CI: 1.029–1.531, *p* = 0.025), and allele T (AOR: 1.185, 95% CI: 1.004–1.397, *p* = 0.044) yielded higher ratios in the non-proliferative diabetic retinopathy (NPDR) subgroup than in the non-DR group. Furthermore, the interval of diabetes mellitus (DM) was significantly shorter in the *LINC00673* SNP rs11655237 CT + TT variant than that in the *LINC00673* SNP rs11655237 wild type (10.44 ± 7.10 vs. 12.98 ± 8.34, *p* = 0.009). In conclusion, the *LINC00673* SNP rs11655237 T allele is associated with a higher probability of NPDR development. Patients with the *LINC00673* SNP rs11655237 CT + TT variant exhibited a short DM interval.

## Introduction

Diabetes mellitus (DM) affects more than 9% of individuals worldwide and can lead to various vascular disorders (Zheng et al., 2018). Diabetic retinopathy (DR) is a complication of DM that is characterized by vascular damage and fluid leakage in the retina. DR can be further divided into non-proliferative diabetic retinopathy (NPDR) and proliferative diabetic retinopathy (PDR), according to the presence of retinal neovascularization (Antonetti et al., 2012). Advanced PDR can cause other ocular diseases, including tractional retinal detachment, vitreous hemorrhage, and neovascular glaucoma (Cheung et al., 2010; Sabanayagam et al., 2019). A previous study estimated that nearly 2.5 and 1.4% of the global cases of blindness and moderate-to-severe visual impairment, respectively, resulted from DR (Flaxman et al., 2017).

The etiology of DR development is multifactorial (Cheung et al., 2010; Antonetti et al., 2012). Apart from DM duration and the condition of blood sugar control (defined as the concentration of glycated hemoglobin [HbA1c] (Sabanayagam et al., 2019; Ghamdi, 2020), biomarkers can also influence the occurrence of DR (Jenkins et al., 2015). Inflammatory cytokines such as interleukins are involved in the development of DR and diabetic macular edema (Capitão and Soares, 2016). Another prominent component in the development of DR is vascular endothelial growth factor (VEGF) (Zhang et al., 2009), which is secreted by the retina during DR progression (Pe’er et al., 1996). In addition, some genes and their variants can influence DR occurrence, including the single nucleotide polymorphisms (SNPs) of VEGF and aquaporin-4 (Khan et al., 2020; Zhou et al., 2021).

Long noncoding RNAs (lncRNAs) are a group of RNAs that could contribute to angiogenesis and cell proliferation in tumor cells (Su S.-C. et al., 2018; Zhao et al., 2020; Su et al., 2021b; Ding et al., 2021). Among this group, long intergenic noncoding RNA 00673 (LINC00673) is associated with several malignancies (Zhu K. et al., 2021; Zhu Y. et al., 2021). In addition, *LINC00673* SNPs can influence the expression of cancers such as oral cancer and gastric cancer (Zhao et al., 2019; Su et al., 2021a). Nevertheless, research on the relationship between the SNPs of *LINC00673* and DR development is rare. Because LINC00673 is downregulated in patients with DR (Cheng et al., 2021), the SNPs of *LINC00673* may also affect the clinical features of DR.

The purpose of this study was to assess the possible correlation between *LINC00673* SNPs (rs6501551, rs9914618, and rs11655237) and the clinical characteristics of DR, including NPDR and PDR. The different phenotypes of *LINC00673* SNP rs11655237 were also separately analyzed.

## Materials and Methods

### Ethical Declarations

This study, conducted in accordance with the 1964 Declaration of Helsinki and its later amendments, was approved by the Institutional Review Board of Chung Shan Medical University Hospital (project identification code: CS1-20048, approval date: 27 April 2020). Written informed consent was signed by the participants.

### Patient Selection

Our case–control study was conducted at Chung Shan Medical University Hospital. A total of 730 patients diagnosed as having DM were included. Among these, 454 patients were categorized as the non-DR group and the other 276 patients were categorized as the DR group, according to fundus examination records in the ophthalmic department. The development of DR was regarded as any of the following fundus findings: dot- and flame-shaped hemorrhage, cotton-wool spot, hard exudate, venous beading, microaneurysm, or intraretinal microvascular abnormality. In the DR group, 111 patients were classified as the PDR group according to any of the following ophthalmological findings: neovascularization of the retina or optic disc, tractional retinal detachment, vitreous hemorrhage, or neovascular glaucoma. The remaining 165 patients constituted the NPDR group.

### Data and Sample Collection

We reviewed the medical records of patients with DM in Chung Shan Medical University Hospital and collected basic data, including age, body mass index, sex, blood sugar level expressed as HbA1c, DM duration, blood pressure, lipid profiles, renal function, and insulin treatment regimen. To analyze *LINC00673* polymorphisms, we used the methods described in our previous study (Hsieh et al., 2021). First, venous blood was drawn from each patient, and the collected blood samples were stored in separate ethylenediaminetetraacetic acid–containing tubes (Hsiao et al., 2010). Next, the blood samples were centrifuged promptly and stored in a laboratory refrigerator at approximately −80°C. Patients were excluded from the study if the genomes in their blood samples had been degraded prior to any analysis.

### DNA Extraction and Determination of *LINC00673* SNP Using Real-Time PCR

A total of three *LINC00673* SNPs, namely, rs6501551 (A/G), rs9914618 (G/A), and rs11655237 (C/T), were selected for phenotype analyses because the minor allele frequencies of these SNPs were higher than 5% and our preceding research indicated the effect of these SNPs on other cancers (Su et al., 2021a). The procedures for DNA extraction and phenotype analyses in this study were in accordance with those of our previous study (Su S. C. et al., 2018). First, DNA was extracted from leukocytes by using QIAamp DNA kits (QIAGEN, Valencia, CA, United States), according to the manufacturer’s instruction for DNA isolation (Chung et al., 2011). Next, the DNA was preserved at approximately −20°C. Genetic polymorphisms of the *LINC00673* SNPs, namely, rs6501551 (A/G) (ID: C_29084653_10), rs9914618 (G/A) (ID: C_29971800_10), and rs11655237 (C/T) (ID: C_345893_20), were then determined using the ABI StepOne Real-Time PCR System (Applied Biosystems, Foster City, CA, United States). The final results of the *LINC00673* genetic polymorphisms were analyzed using SDS version 3.0 software (Applied Biosystems).

### Statistical Analysis

We used SAS version 9.4 (SAS Institute Inc., Cary, NC, United States) for all statistical analyses. Descriptive statistics such as mean, standard deviation, and percentage were used to compare demography, blood sugar status, and other laboratory data between the DR and non-DR groups. An independent *t-*test was used to evaluate the difference of these data between both groups. Next, multiple logistic regression models were applied to evaluate adjusted odds ratios (AORs) and 95% confidence intervals (CIs) of the *LINC00673* SNP distribution and the allele distribution between the two groups. The multiple logistic regressions were adjusted for age, DM duration, insulin treatment, HbA1c, glomerular filtration rate, serum creatinine levels, and HDL cholesterol levels. The same multiple logistic regression models were used in the subgroup analyses for SNP distributions between the non-DR group and each of the NPDR and PDR subgroups. Furthermore, differences in clinicopathological characteristics for each SNP rs11655237 phenotype (i.e., CC and CT + TT) in the DR group were again analyzed using the independent *t-*test. A *p*-value less than 0.05 was considered statistically significant.

## Results

### Characteristics Between the Non-DR and DR Groups

The mean age of the DR group was 62.61 ± 10.72 years, which was significantly higher than that of the non-DR group (60.22 ± 11.22, *p* = 0.005). Parameters related to DM, including DM duration, HbA1c level, and ratio of insulin treatment, were all higher in the DR group (all *p* < 0.001). Serum creatinine was also higher in the DR group (*p* < 0.001), although the glomerular filtration rate (*p* < 0.001) and HDL cholesterol (*p* = 0.017) were lower than those of the non-DR group. The remaining parameters were not significantly different between both groups (all *p* > 0.05) (Table 1).

**TABLE 1:** Clinical and laboratory characteristics of patients with diabetic retinopathy and no diabetic retinopathy.

Variable	Non-DR group (*N* = 454)	DR group (*N* = 276)	*p*-value
Age (years)	60.22 ± 11.22	62.61 ± 10.72	0.005^a^
Male gender [n (%)]	240 (52.9%)	152 (55.1%)	0.562
Duration of DM (years)	9.38 ± 7.03	11.98 ± 7.96	<0.001^a^
HbA1c [% (mmol/mol)]	6.96 ± 0.99	7.59 ± 1.42	<0.001^a^
Insulin treatment [n (%)]	105 (23.1%)	129 (46.7%)	<0.001^a^
Body mass index [kg/m^2^]	26.15 ± 4.32	25.99 ± 4.29	0.631
Systolic blood pressure [mm Hg]	135.44 ± 15.32	137.34 ± 17.37	0.123
Diastolic blood pressure [mm Hg]	76.44 ± 11.27	75.74 ± 11.58	0.419
Serum creatinine [mg/dL]	0.89 ± 0.35	1.55 ± 1.84	<0.001^a^
Glomerular filtration rate [ml/min]	78.44 ± 27.69	62.88 ± 34.15	<0.001^a^
Total cholesterol [mmol/L]	160.52 ± 43.04	165.27 ± 47.61	0.172
HDL cholesterol [μmol/L]	46.29 ± 12.64	43.87 ± 13.58	0.017^a^
LDL cholesterol [μmol/L]	86.60 ± 28.22	86.35 ± 32.86	0.918
Triglycerides, [μmol/L]	140.19 ± 164.95	156.80 ± 117.95	0.154

N, number; DR, diabetic retinopathy; DM, diabetes mellitus; HbA1c, glycated hemoglobin.

a denotes significant difference between the two groups.

### *LINC00673* SNP Distribution Among Different DR Groups

The genotyping frequencies of each *LINC00673* SNP between the DR and non-DR groups are shown in Table 2. No significant differences were observed regarding the distribution of the *LINC00673* SNPs and their alleles between both groups (all *p* > 0.05) (Table 2). In the subgroup analyses, the *LINC00673* SNP rs11655237 CT genotype (AOR: 1.592, 95% CI: 1.059–2.395, *p* = 0.026), *LINC00673* SNP rs11655237 CT + TT genotype (AOR: 1.255, 95% CI: 1.029–1.531, *p* = 0.025), and *LINC00673* SNP rs11655237 allele T (AOR: 1.185, 95% CI: 1.004–1.397, *p* = 0.044) exhibited higher ratios in the NPDR subgroup than in the non-DR group (Table 3). However, no significant differences in genotype frequencies were observed between the PDR subgroup and the non-DR group (all *p* > 0.05; Table 4).

**TABLE 2:** Adjusted odds ratio and 95% confidence intervals of diabetic retinopathy associated with *LINC00673* genotypic frequencies.

Variable	Non-DR group (*N* = 454)	DR group (*N* = 276)	AOR (95% CI)	*p*-value
rs6501551				
AA	334 (73.6%)	200 (72.5%)	1.000 (references)	—
AG	113 (24.9%)	70 (25.4%)	1.032 (0.699–1.525)	0.874
GG	7 (1.5%)	6 (2.1%)	1.836 (0.567–5.945)	0.311
AG + GG	120 (26.4%)	76 (27.5%)	1.038 (0.859–1.254)	0.699
Allele-A	781 (86.0%)	470 (85.1%)	1.000 (references)	—
Allele-G	127 (14.0%)	82 (14.9%)	1.055 (0.892–1.247)	0.533
rs9914618				
GG	302 (66.5%)	173 (62.7%)	1.000 (references)	—
GA	134 (29.5%)	90 (32.6%)	1.065 (0.734–1.544)	0.740
AA	18 (4.0%)	13 (4.7%)	0.932 (0.408–2.131)	0.868
GA + AA	152 (33.5%)	103 (37.3%)	1.023 (0.857–1.222)	0.801
Allele-G	738 (81.3%)	436 (79.0%)	1.000 (references)	—
Allele-A	170 (18.7%)	116 (21.0%)	1.010 (0.869–1.174)	0.894
rs11655237				
CC	301 (66.3%)	167 (60.5%)	1.000 (references)	—
CT	138 (30.4%)	97 (35.1%)	1.296 (0.899–1.869)	0.164
TT	15 (3.3%)	12 (4.4%)	1.655 (0.691–3.967)	0.258
CT + TT	153 (33.7%)	109 (39.5%)	1.153 (0.967–1.376)	0.112
Allele-C	740 (81.5%)	431 (78.1%)	1.000 (references)	—
Allele-T	168 (18.5%)	121 (21.9%)	1.134 (0.978–1.315)	0.096

N, number; DR, diabetic retinopathy; AOR, adjusted odds ratio, estimated by multiple logistic regression models after controlling for age, the duration of DM, HbA1c, insulin treatment, serum creatinine levels, glomerular filtration rate, and HDL cholesterol levels; CI, confidence interval.

**TABLE 3:** Adjusted odds ratio and 95% confidence intervals of non-proliferative diabetic retinopathy associated with *LINC00673* genotypic frequencies.

Variable	Non-DR group (*N* = 454)	NPDR subgroup (*N* = 165)	AOR (95% CI)	*p*-value
rs6501551				
AA	334 (73.6%)	114 (69.1%)	1.000 (references)	—
AG	113 (24.9%)	45 (27.3%)	1.139 (0.731–1.773)	0.565
GG	7 (1.5%)	6 (3.6%)	2.890 (0.903–9.251)	0.074
AG + GG	120 (26.4%)	51 (30.9%)	1.114 (0.901–1.377)	0.320
Allele-A	781 (86.0%)	273 (82.7%)	1.000 (references)	—
Allele-G	127 (14.0%)	57 (17.3%)	1.141 (0.949–1.372)	0.161
rs9914618				
GG	302 (66.5%)	107 (64.8%)	1.000 (references)	—
GA	134 (29.5%)	48 (29.1%)	0.928 (0.600–1.436)	0.738
AA	18 (4.0%)	10 (6.1%)	1.257 (0.527–2.998)	0.607
GA + AA	152 (33.5%)	58 (35.2%)	0.986 (0.803–1.211)	0.895
Allele-G	738 (81.3%)	262 (79.4%)	1.000 (references)	—
Allele-A	170 (18.7%)	68 (20.6%)	1.010 (0.850–1.200)	0.912
rs11655237				
CC	301 (66.3%)	91 (55.2%)	1.000 (references)	—
CT	138 (30.4%)	68 (41.2%)	1.592 (1.059–2.395)	0.026^a^
TT	15 (3.3%)	6 (3.6%)	1.414 (0.492–4.070)	0.520
CT + TT	153 (33.7%)	74 (44.8%)	1.255 (1.029–1.531)	0.025^a^
Allele-C	740 (81.5%)	250 (75.8%)	1.000 (references)	—
Allele-T	168 (18.5%)	80 (24.2%)	1.185 (1.004–1.397)	0.044^a^

N, number; DR, diabetic retinopathy; NPDR, non-proliferative diabetic retinopathy; AOR, adjusted odds ratio, estimated by multiple logistic regression models after controlling for age, the duration of DM, HbA1c, insulin treatment, serum creatinine levels, glomerular filtration rate, and HDL cholesterol levels; CI: confidence interval.

a denotes significant difference about the distribution of polymorphism between the two groups.

**TABLE 4:** Adjusted odds ratio and 95% confidence intervals of proliferative diabetic retinopathy associated with *LINC00673* genotypic frequencies.

Variable	Non-DR group (*N* = 454)	PDR subgroup (*N* = 111)	AOR (95% CI)	*p*-value
rs6501551				
AA	334 (73.6%)	86 (77.5%)	1.000 (references)	—
AG	113 (24.9%)	25 (22.5%)	0.784 (0.433–1.418)	0.421
GG	7 (1.5%)	0 (0.0%)	—	—
AG + GG	120 (26.4%)	25 (22.5%)	0.857 (0.637–1.151)	0.304
Allele-A	781 (86.0%)	197 (88.7%)	1.000 (references)	—
Allele-G	127 (14.0%)	25 (11.3%)	0.844 (0.643–1.108)	0.223
rs9914618				
GG	302 (66.5%)	66 (59.5%)	1.000 (references)	—
GA	134 (29.5%)	42 (37.8%)	1.499 (0.885–2.539)	0.132
AA	18 (4.0%)	3 (2.7%)	0.594 (0.133–2.653)	0.495
GA + AA	152 (33.5%)	45 (40.5%)	1.171 (0.906–1.514)	0.227
Allele-G	738 (81.3%)	174 (78.4%)	1.000 (references)	—
Allele-A	170 (18.7%)	48 (21.6%)	1.082 (0.871–1.343)	0.478
rs11655237				
CC	301 (66.3%)	76 (68.5%)	1.000 (references)	—
CT	138 (30.4%)	29 (26.1%)	0.756 (0.420–1.360)	0.350
TT	15 (3.3%)	6 (5.4%)	2.295 (0.749–7.033)	0.146
CT + TT	153 (33.7%)	35 (31.5%)	0.944 (0.720–1.238)	0.677
Allele-C	740 (81.5%)	181 (81.5%)	1.000 (references)	—
Allele-T	168 (18.5%)	41 (18.5%)	1.026 (0.818–1.287)	0.824

N, number; DR, diabetic retinopathy; PDR: proliferative diabetic retinopathy; AOR, adjusted odds ratio, estimated by multiple logistic regression models after controlling for age, the duration of DM, HbA1c, insulin treatment, serum creatinine levels, glomerular filtration rate, and HDL cholesterol levels; CI: confidence interval.

### Clinical Characteristics and Distribution of *LINC00673* SNP rs11655237 in the DR Group

In the DR group, DM duration was significantly shorter in the *LINC00673* SNP rs11655237 CT + TT variant than in the *LINC00673* SNP rs11655237 wild type (10.44 ± 7.10 versus 12.98 ± 8.34, *p* = 0.009). Other factors, including HbA1c, renal function, and lipid profile, produced similar values between the *LINC00673* SNP rs11655237 wild type and the *LINC00673* SNP rs11655237 CT + TT variant (all *p* > 0.05; Table 5).

**TABLE 5:** Clinical characteristics of diabetic retinopathy individuals according to *LINC00673* rs11655237 genotypes.

Variable	*LINC00673* rs11655237		
	CC (*N* = 167)	CT + TT (*N* = 109)	*p*-value
Duration of DM (years)	12.98 ± 8.34	10.44 ± 7.10	0.009^a^
HbA1c [% (mmol/mol)]	7.64 ± 1.39	7.51 ± 1.46	0.482
Serum creatinine [mg/dL]	1.45 ± 1.40	1.68 ± 2.36	0.318
Glomerular filtration rate [ml/min]	61.73 ± 32.96	64.63 ± 35.99	0.497
Total cholesterol [mmol/L]	161.43 ± 47.95	171.13 ± 46.72	0.108
HDL cholesterol [μmol/L]	42.88 ± 13.06	45.37 ± 14.27	0.148
LDL cholesterol [μmol/L]	85.24 ± 34.07	88.05 ± 31.00	0.502
Triglycerides, [μmol/L]	153.08 ± 109.31	162.46 ± 130.37	0.532

LINC00673, long intergenic noncoding RNA 00673; N, number; DM, diabetes mellitus; HbA1c, glycated hemoglobin.

a denotes significant difference between the two groups.

## Discussion

In this study, the *LINC00673* SNP rs11655237 CT and CT + TT phenotypes and the *LINC00673* SNP rs11655237 T allele were more common in patients with NPDR than in the non-DR population. In addition, *LINC00673* SNP rs11655237 CT + TT variants were associated with a shorter DM interval in the DR group. However, the distribution of the *LINC00673* SNP rs11655237 variant was similar between the PDR and non-DR groups.

Several biochemical pathways are correlated with the occurrence of NPDR and PDR (Jenkins et al., 2015), in which inflammatory growth, and angiogenic molecules play major roles (Jenkins et al., 2015). In the development of DR, hypoxic and ischemic retinas secrete inflammatory cytokines such as interleukin-6 and monocyte chemotactic protein, resulting in a prominent inflammatory reaction and leading to vascular damage and retinal ganglion cell death (Wang and Lo, 2018). VEGF concentration is also elevated in such conditions (Huang et al., 2015). If DR progresses, the retinal concentration of VEGF further increases, and retinal neovascularization eventually develops (Osaadon et al., 2014). Anti-VEGF is the main treatment for PDR and its related complications such as vitreous hemorrhage and neovascular glaucoma (Wang and Lo, 2018). Intraocular steroid injection can also retard the progression of PDR (Iglicki et al., 2018). Regarding genetic polymorphisms, VEGF polymorphism influences the development of DR (Khan et al., 2020). In previous studies, VEGF SNP-634G > C was associated with a higher rate of DR (Qiu et al., 2013), and VEGF SNP rs2146323 was related to DR severity (Churchill et al., 2008). SNP rs11567245 of inflammatory cytokine interleukin-10 could also promote DR development (Shi et al., 2019).

SNPs of lncRNAs have been proposed to influence certain types of malignancies and angiogenic diseases (Peng et al., 2017; Yuan et al., 2019; Weng et al., 2020; Ding et al., 2021; Wang et al., 2021). In previous studies, lncRNA *CCAT1* SNP rs67085638 was correlated with the development of gastric cancer (Olesiński et al., 2021), and lncRNA *PCAT1* and its SNP rs2632159 were associated with a higher rate of colon cancer, which involves an angiogenesis process (Yang et al., 2019). LINC00673, a member of the lncRNA family, can enhance the development of certain neoplasms because of its chromatin modulation and transcriptional regulation functions (Lu et al., 2017; Qiao et al., 2019; Hsieh et al., 2021; Huang et al., 2021). *LINC00673* SNP rs11655237 could alter the development of several cancers, including hepatocellular carcinoma, pancreatic cancer, and oral cancer (Li et al., 2020). Because *LINC00673* SNP is related to cellular proliferation and lncRNA SNP is correlated with angiogenesis, *LINC00673* SNP may also lead to DR development, which is characterized by both cell proliferation and angiogenesis. This hypothesis is aided by our study’s results.

Studies that have discussed the possible correlation between *LINC00673* SNP and DR development are rare; only one study has evaluated the relationship between LINC00673 expression and DR (Cheng et al., 2021). In our study, the frequency of *LINC00673* SNPs (i.e., rs6501551, rs9914618, and rs11655237) was not significantly different between the non-DR and DR groups. In the subgroup analyses that divided the DR group into the NPDR and PDR subgroups, the *LINC00673* SNP rs11655237 CT and CT + TT variants were correlated with NPDR development, with a higher AOR. This result may support the relationship between *LINC00673* SNP rs11655237 and NPDR. Conversely, the *LINC00673* SNP rs11655237 TT variant was not significantly correlated with NPDR development. A total of fifteen patients had the *LINC00673* SNP rs11655237 TT phenotype, of which six patients were in each of the non-DR group and NPDR subgroup, accounting for approximately 3% in each group. Such a few number of cases may lead to statistical bias. The analysis of the T allele effect on *LINC00673* SNP rs11655237 revealed a significantly higher *LINC00673* SNP rs11655237 T-allele distribution in the NPDR subgroup. Consequently, we speculated that the existence of the T allele in *LINC00673* SNP rs11655237, whether in the homogenous or heterogeneous form, is crucial for the development of NPDR. On the contrary, the distribution of the three *LINC00673* SNPs was not significantly different between the non-DR group and the PDR population. It may be possible that the effect of a single SNP is not adequate to trigger the angiogenesis process that contributes to PDR development. Whether the development of PDR is influenced by the LINC00673 SNP in combination with VEGF SNP or other biomarkers requires additional investigation.

Concerning clinicopathological characteristics, our study demonstrated that individuals who had DR with the *LINC00673* SNP rs11655237 CT and TT variants exhibited a significantly shorter DM duration than that with the *LINC00673* SNP rs11655237 CC phenotype. We believe this is preliminary evidence that genetic polymorphisms of *LINC00673* may affect the course of DR development in patients with DM. The DM duration in patients with the *LINC00673* SNP rs11655237 CT and TT variants was nearly 2.5 years shorter than that of patients with the *LINC00673* SNP rs11655237 wild type and also shorter than the mean DM duration in a previous study (Gverović Antunica et al., 2019). Although the exact mechanism underlying this finding is unknown, we speculate that the *LINC00673* SNP rs11655237 CT and TT variants might make the retina more vulnerable to hyperglycemic conditions, even with numerically lower HbA1c levels. This finding, along with the higher genotype frequency of *LINC00673* SNP rs11655237 variants in the NPDR subgroup, may explain the prominent effect of the *LINC00673* SNP rs11655237 variant on NPDR development.

Regarding demographic data and laboratory findings, patients with DR were significantly older than those without DR. Because age is an established risk factor for DR occurrence (Antonetti et al., 2012), our findings agree with those of previous studies (Antonetti et al., 2012). In addition, longer DM duration, higher serum HbA1c concentration, and higher ratio of insulin treatment were observed in the DR group, which could indicate that inadequate blood sugar control is a prominent risk factor for DR (Cheung et al., 2010). Renal function was significantly worse in the DR group. In previous studies, poorly controlled DM was associated with a higher rate of chronic kidney disease (Tziomalos and Athyros, 2015). Chronic kidney disease would in turn contribute to retinal diseases such as retinal microangiopathy and central serous chorioretinopathy (Chang et al., 2019; Kasumovic et al., 2020). It is therefore reasonable that kidney impairment and DR developed concurrently in our study. Higher HDL cholesterol levels in the DR group may have not been clinically significant because both values belonged to the low-HDL status (März et al., 2017).

Our study had several limitations. First, because our study used a case–control design, rather than a cohort design, we could not evaluate the possible causal relationship between the *LINC00673* SNP and the clinical course of DR. Second, some patients may have received DR-related treatment elsewhere, which may have modified their clinical course toward NPDR and could have caused a wrong estimation of the effect of *LINC00673* SNP on PDR development. Additionally, the interval between the internal medicine department visit and the ophthalmic department visit was uneven among the participants.

## Conclusion

In conclusion, the existence of the *LINC00673* SNP rs11655237 T allele is associated with a higher probability of NPDR development. The *LINC00673* SNP rs11655237 variant is correlated with a shorter DM interval in patients with DR; consequently, genetic screening might be suggested for patients with DM. Patients with this genetic polymorphism should observe stricter blood sugar control. Further large-scale prospective studies to examine whether the *LINC00673* polymorphism would interact with the VEGF polymorphism in DR are warranted.

## Data Availability

The original contributions presented in the study are included in the article/Supplementary Material; further inquiries can be directed to the corresponding author.
